# Poly[[[diaqua­cobalt(II)]-bis­[μ_2_-1,1′-(butane-1,4-di­yl)diimidazole-κ^2^ *N* ^3^:*N* ^3′^]] dinitrate]

**DOI:** 10.1107/S1600536809005881

**Published:** 2009-02-25

**Authors:** Yu Su, Chuan He, Zhi-Zhong Sun, Guang-Feng Hou, Jin-Sheng Gao

**Affiliations:** a College of Chemistry and Materials Science, Heilongjiang University, Harbin 150080, People’s Republic of China; b School of Resources and Safety Engineering, China University of Mining and Technology (Beijing Campus), Beijing 100083, People’s Republic of China

## Abstract

In the title compound, {[Co(C_10_H_14_N_4_)_2_(H_2_O)_2_](NO_3_)_2_}_*n*_, the Co^II^ ion lies on an inversion center and is six-coordinated in an octa­hedral environment by four N atoms from four different 1,1′-butane-1,4-diyldiimidazole ligands and two O atoms from the two water mol­ecules. The Co^II^ atoms are bridged by ligands, generating a two-dimensional (4,4)-network. Adjacent fishnet planes are linked to the nitrate anions *via* O—H⋯O hydrogen bonds, forming a three-dimensional supra­molecular structure.

## Related literature

For the synthesis of 1,1′-butane-1,4-diyldiimidazole, see: Ma *et al.* (2003 ▶); Yu *et al.* (2008 ▶) For a related Co complex, see: Dong & Zhang (2006 ▶). 
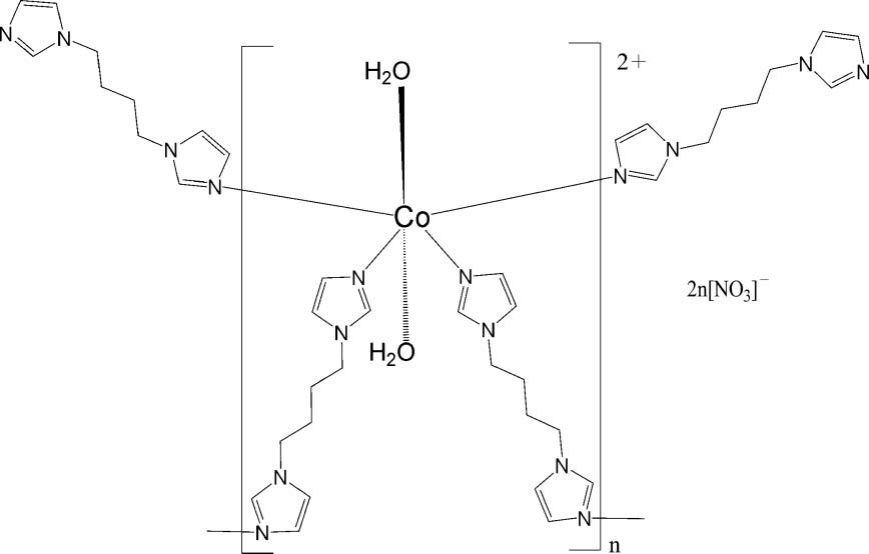


## Experimental

### 

#### Crystal data

[Co(C_10_H_14_N_4_)_2_(H_2_O)_2_](NO_3_)_2_
*M* *_r_* = 599.49
Triclinic, 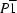

*a* = 8.574 (7) Å
*b* = 8.692 (6) Å
*c* = 9.666 (5) Å
α = 104.71 (2)°
β = 97.14 (3)°
γ = 98.89 (3)°
*V* = 678.2 (8) Å^3^
*Z* = 1
Mo *K*α radiation
μ = 0.70 mm^−1^
*T* = 291 K
0.45 × 0.28 × 0.26 mm

#### Data collection

Rigaku R-AXIS RAPID diffractometer
Absorption correction: multi-scan (*ABSCOR*; Higashi, 1995 ▶) *T*_min_ = 0.745, *T*_max_ = 0.842
6717 measured reflections
3073 independent reflections
2888 reflections with *I* > 2σ(*I*)
*R*_int_ = 0.015

#### Refinement

*R*[*F* ^2^ > 2σ(*F* ^2^)] = 0.031
*wR*(*F* ^2^) = 0.096
*S* = 1.16
3073 reflections
178 parameters
H-atom parameters constrained
Δρ_max_ = 0.35 e Å^−3^
Δρ_min_ = −0.22 e Å^−3^

### 

Data collection: *RAPID-AUTO* (Rigaku, 1998 ▶); cell refinement: *RAPID-AUTO*; data reduction: *CrystalStructure* (Rigaku/MSC, 2002 ▶); program(s) used to solve structure: *SHELXS97* (Sheldrick, 2008 ▶); program(s) used to refine structure: *SHELXL97* (Sheldrick, 2008 ▶); molecular graphics: *SHELXTL* (Sheldrick, 2008 ▶); software used to prepare material for publication: *SHELXL97*.

## Supplementary Material

Crystal structure: contains datablocks global, I. DOI: 10.1107/S1600536809005881/ng2547sup1.cif

Structure factors: contains datablocks I. DOI: 10.1107/S1600536809005881/ng2547Isup2.hkl

Additional supplementary materials:  crystallographic information; 3D view; checkCIF report

## Figures and Tables

**Table:** 

Co1—N3	2.109 (2)
Co1—N1	2.1697 (18)
Co1—O1	2.1838 (16)

**Table:** 

N3—Co1—N1	86.99 (7)
N3—Co1—O1	90.67 (7)
N1—Co1—O1	89.79 (6)

**Table 2:** Hydrogen-bond geometry (Å, °)

*D*—H⋯*A*	*D*—H	H⋯*A*	*D*⋯*A*	*D*—H⋯*A*
O1—H15⋯O4^i^	0.85	1.94	2.775 (3)	167
O1—H16⋯O2^ii^	0.85	2.09	2.930 (3)	171

Symmetry codes: (i) 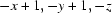; (ii) 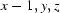.

**Fig. 1.**
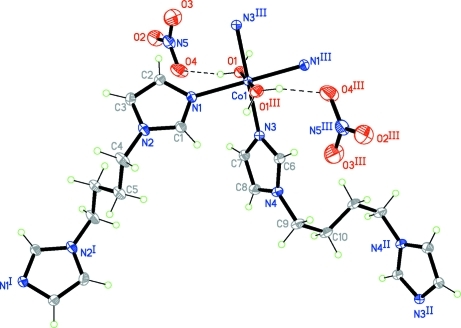
The molecular structure of (I), showing displacement ellipsoids at the 30% probability level for non-H atoms. Dashed lines indicate the hydrogen-bonding interactions [Symmetry code; (I) -x + 1, -y, -z + 1; (II) -x + 1, -y + 2, -z + 2: (III) -x, -y + 1, -z + 1]

**Fig. 2.**
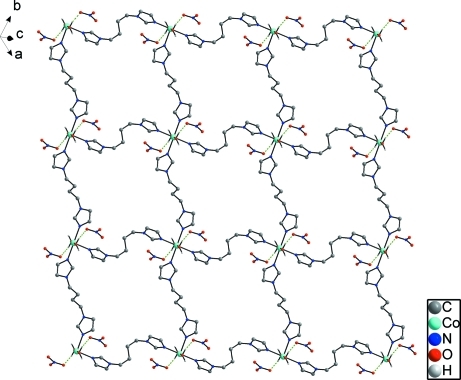
A partial packing view, showing the two-dimensional (4,4)-network. Dashed lines indicate the hydrogen-bonding interactions and no involving H atoms have beeb omitted.

**Fig. 3.**
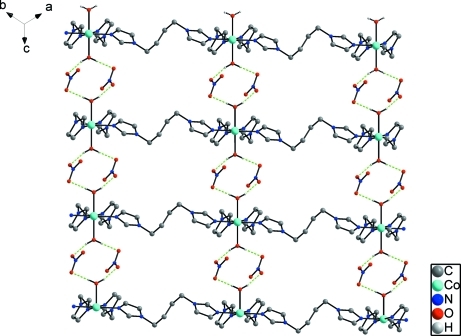
A Partial packing view, shoving the three-dimensional supramolecular structure. Dashed lines indicate the hydrogen-bonding interactions and no involving H atoms have beeb omitted.

## supplementary crystallographic information

### Comment

The 1,1'-butane-1,4-diyldiimidazole as a flexible ligand exhibit a variety of supramolecular aggregation patterns (Ma *et al.*, 2003; Dong *et al.*, 2006; Yu *et al.*, 2008). In this paper, we report the new title compound, (I), synthssized by the reaction of 1,1'-butane-1,4-diyldiimidazole ligand and cobalt dinitrate in aqua solution.

In (I), each Co^II^ atom is located on a inversion centre and is six-coordinated in an octahedral environment by four N atoms from four different 1,1'-butane-1,4-diyldiimidazole ligands and two O atoms form the two water molecules (Fig. 1). The Co—N and Co—O distances are normal (Table 1). The Co^II^ atoms are bridged by ligands, generating a two-dimensional (4,4)-network (Fig. 2).

In the crystal, a *R*_4_^4^(12) motif is built up by O—H···O hydrogen bonding interaction between the uncoordinated nitrate anions and the coordinated water molecules,which linke the adjacent fishnet planes to a three-dimensional supramolecular structure (Fig. 3, Table 2).

### Experimental

1,1'-Butane-1,4-diyldiimidazole ligand was prepared from imidazole and 1,4-dibromobutane in DMSO (Ma *et al.*, 2003*a*). 1,1'-Butane-1,4-diyldiimidazole (0.76 g, 4 mmol) and cobalt dinitrate (0.73 g, 4 mmol) were dissolved in hot aqua solution (10 ml) to give a clear solution. The resulting solution was allowed to stand in a desiccator at room temperature for a week, pink crystals of (I) were obtained.

### Refinement

H atoms bound to C atoms were placed in calculated positions and treated as riding on their parent atoms, with C—H = 0.93 Å (aromatic), C—H = 0.97 Å (methylene), and with *U*_iso_(H) = 1.2*U*_eq_(C). Water H atoms were initially located in a difference Fourier map, but they were treated as riding on their parent atoms with O—H = 0.85 Å and with with *U*_iso_(H) = 1.5*U*_eq_(O).

### Crystal data

**Table:**

[Co(C_10_H_14_N_4_)_2_(H_2_O)_2_](NO_3_)_2_	*Z* = 1
*M**_r_* = 599.49	*F*(000) = 313
Triclinic, *P*1	*D*_x_ = 1.468 Mg m^−^^3^
Hall symbol: -P 1	Mo *K*α radiation, λ = 0.71073 Å
*a* = 8.574 (7) Å	Cell parameters from 6295 reflections
*b* = 8.692 (6) Å	θ = 3.0–27.5°
*c* = 9.666 (5) Å	µ = 0.70 mm^−^^1^
α = 104.71 (2)°	*T* = 291 K
β = 97.14 (3)°	Block, brown
γ = 98.89 (3)°	0.45 × 0.28 × 0.26 mm
*V* = 678.2 (8) Å^3^	

### Data collection

**Table:**

Rigaku R-AXIS RAPID diffractometer	3073 independent reflections
Radiation source: fine-focus sealed tube	2888 reflections with *I* > 2σ(*I*)
graphite	*R*_int_ = 0.015
ω scans	θ_max_ = 27.5°, θ_min_ = 3.0°
Absorption correction: multi-scan (*ABSCOR*; Higashi, 1995)	*h* = −11→11
*T*_min_ = 0.745, *T*_max_ = 0.842	*k* = −11→11
6717 measured reflections	*l* = −12→12

### Refinement

**Table:**

Refinement on *F*^2^	Primary atom site location: structure-invariant direct methods
Least-squares matrix: full	Secondary atom site location: difference Fourier map
*R*[*F*^2^ > 2σ(*F*^2^)] = 0.031	Hydrogen site location: inferred from neighbouring sites
*wR*(*F*^2^) = 0.096	H-atom parameters constrained
*S* = 1.16	*w* = 1/[σ^2^(*F*_o_^2^) + (0.0539*P*)^2^ + 0.1966*P*] where *P* = (*F*_o_^2^ + 2*F*_c_^2^)/3
3073 reflections	(Δ/σ)_max_ < 0.001
178 parameters	Δρ_max_ = 0.35 e Å^−^^3^
0 restraints	Δρ_min_ = −0.22 e Å^−^^3^

### Special details

**Table:**

Geometry. All e.s.d.'s (except the e.s.d. in the dihedral angle between two l.s. planes) are estimated using the full covariance matrix. The cell e.s.d.'s are taken into account individually in the estimation of e.s.d.'s in distances, angles and torsion angles; correlations between e.s.d.'s in cell parameters are only used when they are defined by crystal symmetry. An approximate (isotropic) treatment of cell e.s.d.'s is used for estimating e.s.d.'s involving l.s. planes.
Refinement. Refinement of *F*^2^ against ALL reflections. The weighted *R*-factor *wR* and goodness of fit *S* are based on *F*^2^, conventional *R*-factors *R* are based on *F*, with *F* set to zero for negative *F*^2^. The threshold expression of *F*^2^ > σ(*F*^2^) is used only for calculating *R*-factors(gt) *etc*. and is not relevant to the choice of reflections for refinement. *R*-factors based on *F*^2^ are statistically about twice as large as those based on *F*, and *R*- factors based on ALL data will be even larger.

### Fractional atomic coordinates and isotropic or equivalent isotropic displacement parameters (Å^2^)

**Table:**

	*x*	*y*	*z*	*U*_iso_*/*U*_eq_	
C1	0.1755 (2)	0.2246 (2)	0.53022 (19)	0.0325 (4)	
H1	0.2106	0.2822	0.6272	0.039*	
C2	0.0680 (2)	0.1572 (2)	0.30656 (19)	0.0336 (4)	
H2	0.0131	0.1605	0.2184	0.040*	
C3	0.1346 (2)	0.0324 (2)	0.3277 (2)	0.0372 (4)	
H3	0.1343	−0.0642	0.2586	0.045*	
C4	0.2858 (2)	−0.0192 (3)	0.5505 (2)	0.0424 (5)	
H4	0.2518	−0.1333	0.4989	0.051*	
H5	0.2548	−0.0033	0.6455	0.051*	
C5	0.4672 (2)	0.0257 (2)	0.5695 (2)	0.0386 (4)	
H6	0.4997	0.1423	0.6092	0.046*	
H7	0.5142	−0.0241	0.6393	0.046*	
C6	0.2528 (2)	0.6569 (2)	0.77832 (18)	0.0304 (3)	
H8	0.1736	0.6650	0.8359	0.036*	
C7	0.3724 (2)	0.6120 (2)	0.59375 (19)	0.0315 (3)	
H9	0.3904	0.5825	0.4985	0.038*	
C8	0.4887 (2)	0.6696 (2)	0.7135 (2)	0.0352 (4)	
H10	0.5989	0.6866	0.7159	0.042*	
C9	0.4832 (3)	0.7684 (3)	0.9839 (2)	0.0437 (5)	
H11	0.5643	0.7091	1.0092	0.052*	
H12	0.4018	0.7584	1.0441	0.052*	
C10	0.5592 (2)	0.9465 (3)	1.0135 (2)	0.0449 (5)	
H13	0.6130	0.9868	1.1137	0.054*	
H14	0.6398	0.9550	0.9523	0.054*	
Co1	0.0000	0.5000	0.5000	0.02274 (11)	
N1	0.09324 (17)	0.27910 (17)	0.43482 (15)	0.0296 (3)	
N2	0.20264 (17)	0.07610 (18)	0.47097 (17)	0.0318 (3)	
N3	0.22398 (16)	0.60381 (16)	0.63499 (15)	0.0273 (3)	
N4	0.41025 (18)	0.69767 (19)	0.83026 (16)	0.0329 (3)	
N5	0.8937 (2)	0.6642 (2)	0.02300 (18)	0.0456 (4)	
O1	0.08701 (16)	0.57801 (16)	0.32103 (13)	0.0362 (3)	
H15	0.1299	0.5143	0.2619	0.054*	
H16	0.0215	0.6177	0.2734	0.054*	
O2	0.8432 (2)	0.6759 (3)	0.13902 (17)	0.0653 (5)	
O3	1.0374 (3)	0.7011 (3)	0.0241 (2)	0.0777 (6)	
O4	0.7986 (3)	0.6117 (3)	−0.09384 (18)	0.0784 (7)	

### Atomic displacement parameters (Å^2^)

**Table:**

	*U*^11^	*U*^22^	*U*^33^	*U*^12^	*U*^13^	*U*^23^
C1	0.0368 (9)	0.0342 (9)	0.0280 (8)	0.0154 (7)	0.0043 (6)	0.0069 (6)
C2	0.0329 (9)	0.0340 (9)	0.0296 (8)	0.0099 (7)	−0.0003 (6)	0.0018 (7)
C3	0.0351 (9)	0.0295 (8)	0.0410 (10)	0.0095 (7)	0.0044 (7)	−0.0016 (7)
C4	0.0378 (10)	0.0439 (10)	0.0616 (12)	0.0199 (8)	0.0163 (9)	0.0325 (9)
C5	0.0347 (9)	0.0401 (10)	0.0481 (11)	0.0173 (8)	0.0071 (8)	0.0188 (8)
C6	0.0275 (8)	0.0326 (8)	0.0281 (8)	0.0071 (6)	0.0016 (6)	0.0040 (6)
C7	0.0294 (8)	0.0352 (9)	0.0289 (8)	0.0104 (7)	0.0043 (6)	0.0050 (6)
C8	0.0252 (8)	0.0408 (9)	0.0361 (9)	0.0074 (7)	0.0022 (7)	0.0052 (7)
C9	0.0417 (10)	0.0535 (12)	0.0264 (9)	0.0104 (9)	−0.0092 (7)	0.0006 (8)
C10	0.0347 (10)	0.0518 (12)	0.0331 (10)	0.0069 (8)	−0.0095 (7)	−0.0068 (8)
Co1	0.02260 (16)	0.02296 (16)	0.02042 (16)	0.00763 (11)	−0.00069 (10)	0.00224 (11)
N1	0.0317 (7)	0.0279 (7)	0.0289 (7)	0.0123 (6)	0.0024 (5)	0.0046 (5)
N2	0.0292 (7)	0.0300 (7)	0.0415 (8)	0.0124 (6)	0.0095 (6)	0.0137 (6)
N3	0.0256 (7)	0.0269 (7)	0.0267 (7)	0.0074 (5)	−0.0003 (5)	0.0034 (5)
N4	0.0286 (7)	0.0367 (8)	0.0273 (7)	0.0071 (6)	−0.0034 (5)	0.0017 (6)
N5	0.0577 (11)	0.0593 (11)	0.0302 (8)	0.0315 (9)	0.0132 (7)	0.0166 (7)
O1	0.0396 (7)	0.0424 (7)	0.0261 (6)	0.0096 (5)	0.0033 (5)	0.0091 (5)
O2	0.0727 (12)	0.0991 (15)	0.0351 (8)	0.0308 (11)	0.0235 (8)	0.0233 (9)
O3	0.0612 (12)	0.1029 (17)	0.0649 (12)	0.0139 (11)	0.0261 (10)	0.0096 (11)
O4	0.0786 (13)	0.1321 (19)	0.0324 (8)	0.0623 (13)	0.0048 (8)	0.0143 (10)

### Geometric parameters (Å, °)

**Table:**

C1—N1	1.318 (2)	C8—N4	1.373 (2)
C1—N2	1.341 (2)	C8—H10	0.9300
C1—H1	0.9300	C9—N4	1.470 (2)
C2—C3	1.350 (3)	C9—C10	1.523 (3)
C2—N1	1.379 (2)	C9—H11	0.9700
C2—H2	0.9300	C9—H12	0.9700
C3—N2	1.366 (3)	C10—C10^ii^	1.521 (4)
C3—H3	0.9300	C10—H13	0.9700
C4—N2	1.469 (2)	C10—H14	0.9700
C4—C5	1.519 (3)	Co1—N3	2.109 (2)
C4—H4	0.9700	Co1—N3^iii^	2.109 (2)
C4—H5	0.9700	Co1—N1^iii^	2.1697 (18)
C5—C5^i^	1.510 (4)	Co1—N1	2.1697 (18)
C5—H6	0.9700	Co1—O1^iii^	2.1838 (16)
C5—H7	0.9700	Co1—O1	2.1838 (16)
C6—N3	1.322 (2)	N5—O3	1.222 (3)
C6—N4	1.339 (2)	N5—O2	1.238 (2)
C6—H8	0.9300	N5—O4	1.243 (3)
C7—C8	1.360 (3)	O1—H15	0.8501
C7—N3	1.377 (2)	O1—H16	0.8500
C7—H9	0.9300		
			
N1—C1—N2	112.01 (16)	C9—C10—H13	108.7
N1—C1—H1	124.0	C10^ii^—C10—H14	108.7
N2—C1—H1	124.0	C9—C10—H14	108.7
C3—C2—N1	110.00 (16)	H13—C10—H14	107.6
C3—C2—H2	125.0	N3—Co1—N3^iii^	180.0
N1—C2—H2	125.0	N3—Co1—N1^iii^	93.01 (7)
C2—C3—N2	106.29 (15)	N3^iii^—Co1—N1^iii^	86.99 (7)
C2—C3—H3	126.9	N3—Co1—N1	86.99 (7)
N2—C3—H3	126.9	N3^iii^—Co1—N1	93.01 (7)
N2—C4—C5	113.21 (16)	N1^iii^—Co1—N1	180.0
N2—C4—H4	108.9	N3—Co1—O1^iii^	89.33 (7)
C5—C4—H4	108.9	N3^iii^—Co1—O1^iii^	90.67 (7)
N2—C4—H5	108.9	N1^iii^—Co1—O1^iii^	89.79 (6)
C5—C4—H5	108.9	N1—Co1—O1^iii^	90.21 (6)
H4—C4—H5	107.8	N3—Co1—O1	90.67 (7)
C5^i^—C5—C4	113.9 (2)	N3^iii^—Co1—O1	89.33 (7)
C5^i^—C5—H6	108.8	N1^iii^—Co1—O1	90.21 (6)
C4—C5—H6	108.8	N1—Co1—O1	89.79 (6)
C5^i^—C5—H7	108.8	O1^iii^—Co1—O1	180.0
C4—C5—H7	108.8	C1—N1—C2	104.72 (15)
H6—C5—H7	107.7	C1—N1—Co1	121.60 (12)
N3—C6—N4	111.57 (16)	C2—N1—Co1	133.01 (12)
N3—C6—H8	124.2	C1—N2—C3	106.97 (15)
N4—C6—H8	124.2	C1—N2—C4	124.90 (17)
C8—C7—N3	109.66 (16)	C3—N2—C4	128.10 (16)
C8—C7—H9	125.2	C6—N3—C7	105.41 (14)
N3—C7—H9	125.2	C6—N3—Co1	127.19 (12)
C7—C8—N4	105.97 (16)	C7—N3—Co1	126.95 (12)
C7—C8—H10	127.0	C6—N4—C8	107.39 (15)
N4—C8—H10	127.0	C6—N4—C9	125.56 (17)
N4—C9—C10	110.98 (17)	C8—N4—C9	126.96 (16)
N4—C9—H11	109.4	O3—N5—O2	119.7 (2)
C10—C9—H11	109.4	O3—N5—O4	120.4 (2)
N4—C9—H12	109.4	O2—N5—O4	119.8 (2)
C10—C9—H12	109.4	Co1—O1—H15	119.0
H11—C9—H12	108.0	Co1—O1—H16	115.0
C10^ii^—C10—C9	114.1 (2)	H15—O1—H16	109.0
C10^ii^—C10—H13	108.7		

Symmetry codes: (i) −*x*+1, −*y*, −*z*+1; (ii) −*x*+1, −*y*+2, −*z*+2; (iii) −*x*, −*y*+1, −*z*+1.

### Hydrogen-bond geometry (Å, °)

**Table:**

*D*—H···*A*	*D*—H	H···*A*	*D*···*A*	*D*—H···*A*
O1—H15···O4^iv^	0.85	1.94	2.775 (3)	167
O1—H16···O2^v^	0.85	2.09	2.930 (3)	171

Symmetry codes: (iv) −*x*+1, −*y*+1, −*z*; (v) *x*−1, *y*, *z*.
